# Physiological Response of Maize Plants (*Zea mays* L.) to the Use of the Potassium Quercetin Derivative

**DOI:** 10.3390/ijms22147384

**Published:** 2021-07-09

**Authors:** Dagmara Migut, Marta Jańczak-Pieniążek, Tomasz Piechowiak, Jan Buczek, Maciej Balawejder

**Affiliations:** 1 Department of Crop Production, University of Rzeszow, Zelwerowicza 4, 35-601 Rzeszow, Poland; mjanczak@ur.edu.pl (M.J.-P.); jbuczek@ur.edu.pl (J.B.); 2 Department of Food Chemistry and Toxicology, University of Rzeszow, Ćwiklińskiej 1A, 35-601 Rzeszów, Poland; tpiechowiak@ur.edu.pl (T.P.); maciejb@ur.edu.pl (M.B.)

**Keywords:** maize, foliar fertilisation, K-quercetin derivative, gas exchange, chlorophyll content, chlorophyll fluorescence, antioxidant capacity

## Abstract

Plant production technologies based solely on the improvement of plants themselves face obstacles resulting from the natural limitations of the biological potential of varieties. Therefore, new substances are sought that positively influence the growth and development of plants and increase resistance to various biotic and abiotic stresses, which also translates into an increase in obtained yields. The exogenous application of various phytoprotectants shows great promise in terms of cost effectiveness compared to traditional breeding methods or transgenic approaches in relation to increasing plant tolerance to abiotic stresses. Quercetin is a strong antioxidant among phenolic compounds, and it plays a physiological and biochemical role in plants. As such, the aim of this research was to assess the effect of an aqueous solution of a quercetin derivative with potassium, applied in various concentrations (0.5%, 1.0%, 3.0% and 5.0%), on the efficiency of the photosynthetic apparatus and biochemical properties of maize. Among the tested variants, compared to the control, the most stimulating effect on the course of physiological processes (P_N_, g_s_, c_i_, CCI, F_v_/F_m_, F_v_/F_0_, PI) in maize leaves was found in 3.0 and 5.0% aqueous solutions of the quercetin derivative. The highest total antioxidant capacity and total content of polyphenolic compounds were found for plants sprayed with 5.0% quercetin derivative solution; therefore, in this study, the optimal concentration could not be clearly selected.

## 1. Introduction

In the 21st century, efficient crops will play a major role in increasing yields compared to the 20th century. This is mainly due to the limited resources of land and water available for plant production, higher costs of inorganic fertilisers used, decreasing trends in yields of selected crops worldwide and increasing environmental concerns [1]. Maize (*Zea mays* L.) is one of the most commonly cultivated plants in the world, and the dynamics of its production are constantly changing [2,3]. The growing interest in cultivating this plant results from the possibility of its universal application as food, animal feed or for industrial or energy plants [4]. Regardless of the direction of maize cultivation, modern agriculture is looking for technological solutions aimed at increasing yields while also improving their quality [5,6].

Abiotic stresses such as extreme temperatures, salinity, drought, nutrient deficiency or excess, metals and metalloids in the soil and UV radiation are among the major threats to agricultural productivity worldwide. Moreover, they can affect almost all metabolic processes in plants [7,8,9,10,11], which are able to detect environmental stimuli and adapt to different environments. However, their degree of tolerance and their ability to adapt to abiotic stresses vary between species and cultivars. Crops exposed to abiotic stress respond by activating defence mechanisms; therefore, crops in the early stage of stress do not show visible symptoms, but their physiology may undergo significant changes [12,13,14,15]. The secondary metabolites produced by a plant regulate its physiological processes to deal with stress. The antioxidant defence system is highly effective in detoxifying overproduced reactive oxygen species (ROS), and consists of enzyme compounds (superoxide dismutase (SOD), catalase (CAT), ascorbate peroxidase (APX), monodehydroascorbate reductase (MDHAR), dehydroascorbate reductase (DHAR), glutathione reductase (GR), glutathione peroxidase (GPX) and glutathione S-transferase (GST)), as well as non-enzymatic components (ascorbate (AsA), glutathione (GSH), carotenoids, phenolic compounds, alkaloids, flavanones and anthocyanins) [16]. However, a plant’s capacity for genetic self-defence is not sufficient to fully it from stress-induced damage.

Therefore, the use of various chemical compounds as protective and stimulating substances has recently become quite popular. Biostimulants containing bioactive compounds can improve plant efficiency in a short period of time, with a lower impact on the environment and lower financial outlays. These compounds improve nutrient use efficiency and may ameliorate the negative effects of abiotic and biotic stresses to some extent [17,18]. In maize cultivation, the use of biostimulants helps to improve physiological processes, which results in obtaining a higher yield [19,20,21]. Among the secondary metabolites, many phenolic compounds confer tolerance to stress, especially flavonoids and phenolic acids, which are produced via the shikimate-phenylpropanoid biosynthetic pathway [22,23]. Currently, research uses various groups of chemical substances as phytoprotectants, including phytohormones, organic acids, antioxidants and other secondary metabolites of plant origin [24,25,26]. Quercetin (3.3′,4′,5.7-pentahydroxyflavone) belongs to a group of flavonoids found mainly in the form of glycosides in plants [27]. Its most important functions are to ensure plant–environment communication and protect plants, mainly through antioxidant activity, i.e., protection photosynthetic machinery against oxidative stress, which can damage the cell’s DNA [28,29]. Moreover, quercetin can chelate transitive metal ions responsible for the production of ROS [30,31,32]. Kobylińska [33] reports that flavonoids (including quercetin) act in the plant as UV protective agents, pollinator attractants and antimicrobial compounds. Moreover, quercetin is a secondary metabolite synthesised in plants during biotic and abiotic stresses [34,35]. Secondary metabolites participate in the light-dependent photosynthesis phase, in which they catalyse electron transport. Moreover, they determine the dynamics of carbon metabolism by changing the rate of accumulation and photophosphorylation of the reducer and by changing the activity of enzymes [33,36,37]. Low doses of exogenous quercetin enhance gluconeogenesis and inhibit glycolysis, which results in a significant increase in monosaccharide content [38,39]. In many in vitro studies and animal models, flavonoids, including quercetin, have been found to have anti-inflammatory, antioxidant, antibacterial, antiviral, hepatoprotective, antiallergic, anticoagulant and immunomodulating effects [40,41,42,43,44].

However, there is little information on its use in plant production, so it has been hypothesised that the K-quercetin derivative can be successfully used as a plant growth promoting substance. The aim of the experiment was to determine whether a quercetin derivative can act as a biostimulant and positively influence the physiological characteristics and growth of plants.

## 2. Results and Discussion

### 2.1. Basic Characteristics of the Potassium Quercetin Derivative

**Figure 1:**
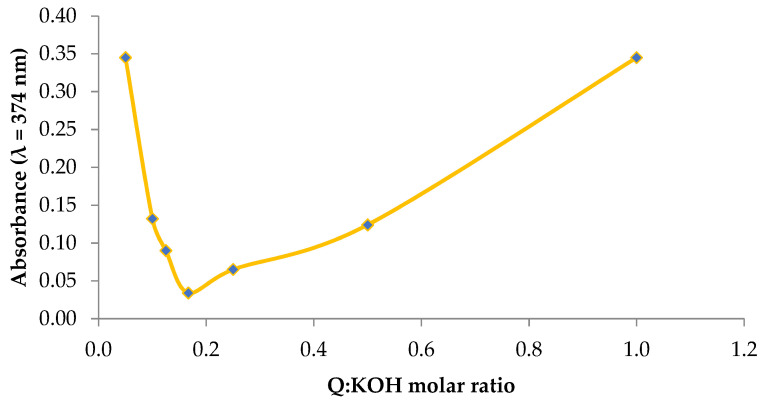
Determination of optimal molar ratios between reactants by Job’s method.

As part of the study, the optimal molar fractions of the reagents in which the potassium quercetin derivative formation reaction proceeds efficiently, and all functional groups that participate in the reaction, were first determined. It was found that the addition of KOH solution to the methanolic quercetin solution causes a significant change in the colour of the solution, which was observed in the form of changes in the absorbance of the solutions (Figure 1). Therefore, Job’s method was used to determine the optimal molar fractions of the reagents. Based on the Job curve, it was found that the optimal molar fraction of quercetin to potassium hydroxide should be 1:7.

**Figure 2:**
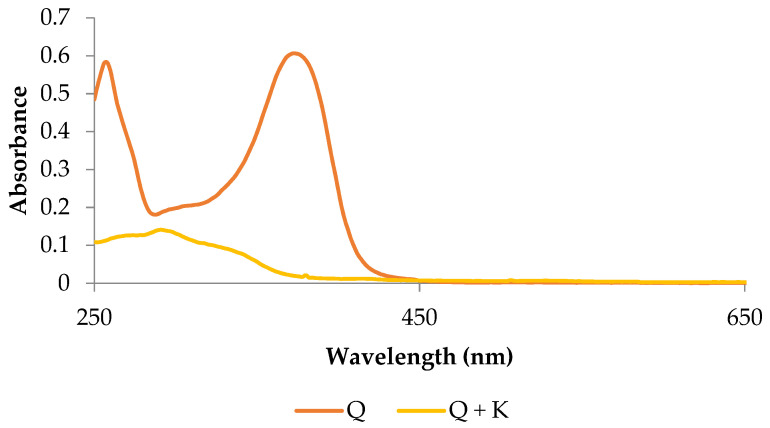
UV-Vis spectrum of potassium quercetin derivative and quercetin: Q—methanolic solution of quercetin with a concentration of 0.1 mg∙mL^−1^; Q + K—methanolic solution of potassium quercetin derivative with a concentration of 0.1 mg∙mL^−1^.

The quercetin derivative was subjected to UV-Vis analysis. On the basis of the obtained spectra, shifts in the position of the absorption maxima for quercetin complexes towards long wavelengths (bathochromic shifts) were noticed, as was a decrease in the absorption intensity (hypsochromic effect) in relation to the quercetin standard, which indicates the formation of derivatives (Figure 2).

The potassium quercetin derivative was then analysed for anti-radical activity against ABTS and DPPH. In the course of the study, it was found that the antioxidant activity against ABTS and DPPH was much lower than the activity of the quercetin standard (Table 1), but the produced potassium derivative was characterised by a higher ABTS and DPPH scavenging ability than ascorbic acid, which was comparable to Trolox.

### 2.2. Gas Exchange

The closure of the stomata is the first response of plants to abiotic stress [45]. Unfavourable environmental conditions allow them to increase the rate of respiration. It is a prerequisite for the production of ATP in order to activate osmotic soluble substances (activate cells under stress), which reduce the osmotic potential of the cell, thus increasing its water uptake [46]. Antioxidants, including quercetin, belong to the group of organic compounds that can play an important role in alleviating stress related to environmental factors, such as drought, high temperature and salinity, through osmotic regulation [36,47], and thus positively affect the course of gas exchange.

**Figure 3:**
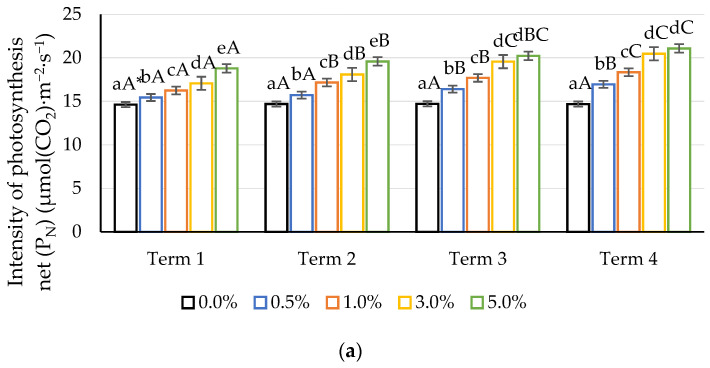

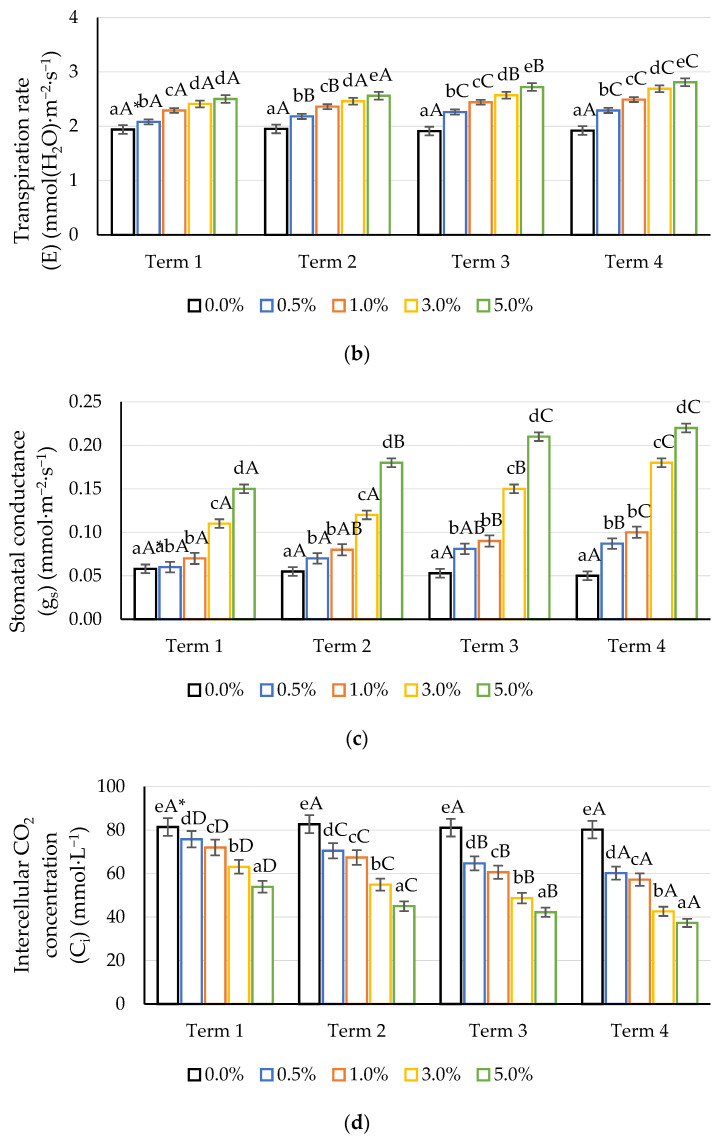
Impact of different aqueous concentrations of potassium quercetin derivative on maize gas exchange parameters: (**a**) net photosynthetic rate (P_N_), (**b**) transpiration rate (E), (**c**) stomatal conductance (g_s_), (**d**) intercellular CO_2_ concentration (C_i_). * Lowercase letters indicate significant differences between the means on respective measurement times, and capital letters indicate significant differences between means on the measurement times for respective potassium quercetin derivative concentrations and exposure times (*n* = 20, *p* < 0.05).

In the conducted study, a significant influence of the applied concentration of quercetin derivative was observed on the parameters of gas exchange in maize leaves (Figure 3). On the first day after the first application (Term 1), there was a significant increase in P_N_, E and g_s_. The beneficial effect of the quercetin derivative on the gas exchange process in maize leaves was also observed on the seventh day after the first application (Term 2). The values of the P_N_, E and g_s_ parameters after the use of 0.5%, 1%, 3% and 5% concentrations were significantly different compared to the control. After the second application of quercetin, a significant increase in P_N_, E and g_s_ was observed compared to the control, both the first day after the second application (Term 3) and seven days after the second application (Term 4). In the case of the C_i_, a decrease in the value of the analysed parameter was observed over the duration of the experiment, regardless of the concentration of the quercetin derivative applied.

All environmental stresses, including the spread of soil and water salinity, the presence of heavy metals in soil or the effects of tropospheric ozone (O_3_), have a destructive effect on agricultural production [9,48,49]; therefore, new methods for reducing the effects of stress and preventing its occurrence are sought. For example, contamination with heavy metals is an increasing problem for the human population due to their potential for biomagnification in plants and subsequent transmission to humans [50,51]. In maize, heavy metals induce many biochemical, morphological and physiological changes that affect normal metabolism, especially photosynthesis [52]. Cadmium (Cd) can reduce the efficiency of light use and significantly damage photosystem I and II [53]. Cd and zinc (Zn) can damage the water-splitting complex and thus reduce the net electron flow to linear electron transport [54]. Cd and Zn also reduce the activity of RuBisCO (ribulose-1,5-bisphosphate carboxylase/oxygenase), which translates into net carbohydrate production by a plant [55]. The decrease in RuBisCO content in maize leaves is one of the reasons for the reduced photosynthetic capacity of plants subjected to stress, and may contribute to toxic premature aging and reduce the CO_2_ binding process in the Calvin–Benson cycle [56]. Bukhari et al. [31] report that hydroxyl groups present in the structure of quercetin have the ability to form complexes with various metal ions, affecting its biological activity; therefore, it can be assumed that quercetin can be used in crops grown in areas threatened by the action of heavy metals. The interaction of flavonoids with metal ions can alter the antioxidant properties and some of the biological effects of flavonoids. They found that the Cu-quercetin complex exhibited higher antioxidant activity compared to pure quercetin. The authors’ own research showed that the K-quercetin complex also showed a higher ABTS and DPPH scavenging ability than ascorbic acid, which was comparable to Trolox.

Additionally, oxidative stress caused by excessive ROS production is a serious threat to photosynthesis [57]. The main source of ROS in plants is photosynthetic dissipation (the dissipation of excess excitation energy absorbed by chlorophyll through various pathways in chloroplasts) in the electron transport chain (ETC) [58]. For example, the action of tropospheric O_3_ activates many signalling pathways that are tightly regulated at many levels [59]. O_3_ enters the apoplast through the stomata, where it is degraded into ROS, causing an increase in the level of calcium in the cytosol, which in turn leads to the closure of the stomata regulated by the activity of ion channels, thus limiting its further penetration [60]. Once a certain level of ROS is reached, extracellular and intracellular signalling pathways are triggered, leading to hormonal, biochemical and transcriptional changes [45]. Similarly, the presence of Cd causes the release of the superoxide radical (O_2_^•−^), hydrogen peroxide (H_2_O_2_) and hydroxyl radical (HO^•^), followed by membrane degradation, while Zn may induce structural and functional changes in the photosynthetic apparatus [61].

The highest concentration (5%) had the most favourable effect on P_N_, E and g_s_. However, during the experiment, one could observe smaller and smaller differences in the values of the analysed parameters between the values obtained at subsequent measurement dates. It seems that the most intense response of plants to foliar application of quercetin can be observed on the first and second measurement dates. After the next application, an increase in the analysed parameters is observed, but it is not as dynamic. This may suggest that the first dose of the derivative has a strong stimulating effect, and the next one may be used to maintain the positive effect of quercetin. The increase in g_s_, found as a result of the action of the quercetin derivative, reduced the accumulation of intracellular CO_2_ in the mesophyll and prompted a decrease in the value of C_i_. This phenomenon was accompanied by an increase in the intensity of P_N_; therefore, it also seems justified to establish a single dose of quercetin in the presence of environmental stress. Longer exposure to the stress factors increases the concentration of C_i_, which indicates a reduction in the ability to bind CO_2_ in the Calvin–Benson cycle. A significant reduction in photosynthesis efficiency may indicate degradation of the photosynthetic apparatus [62]. Quercetin is located in the chloroplast envelope membrane [63,64]. This localization suggests its role in regulating and limiting the intensity of light available to the plant [65]. Flavonoids including quercetin perform multiple photoprotection functions by activating UV-B screening compounds, as well as free-radical scavengers. Quercetin also induces structural changes in thylakoid membranes, which is a possible reason for its protective role in the photosynthetic apparatus [29].

### 2.3. Relative Chlorophyll Content

For many years, agricultural practice in EU countries, which has adapted to introduced directives, has been characterised by the use of environmentally friendly technologies to reduce the use of pesticides and eliminate them from the environment [66]. Consequently, it is becoming more and more challenging for producers to reduce abiotic and biotic stresses in plants. Therefore, it is important to look for new ways to stimulate the physiological processes in plants and to stimulate biological processes in the soil aimed at controlling and accelerating the processes of life, thus increasing the resistance of plants to stressful conditions. It is essential that these substances are safe for the environment.

Chlorophyll is one of the most important biochemical features related to the availability of water and the level of plant nutrition, and it reflects plants’ health status [67,68]. Reduction in chlorophyll content in plants exposed to abiotic stress may result from disintegration of thylakoid membranes, with greater degradation than chlorophyll synthesis through the formation of proteolytic enzymes such as chlorophyllase, which is responsible for chlorophyll degradation, as well as damage to the photosynthetic apparatus [69]. This slows down the rate of photosynthesis in the plant and inhibits ion accumulation [70,71,72]. The use of an aqueous solution of a quercetin derivative can stimulate the tolerance of a plant to abiotic stresses by enhancing antioxidant enzymes and securing photosynthetic activity, as well as preventing membrane peroxidation or strengthening a plant’s defence system against oxidative damage.

**Figure 4:**
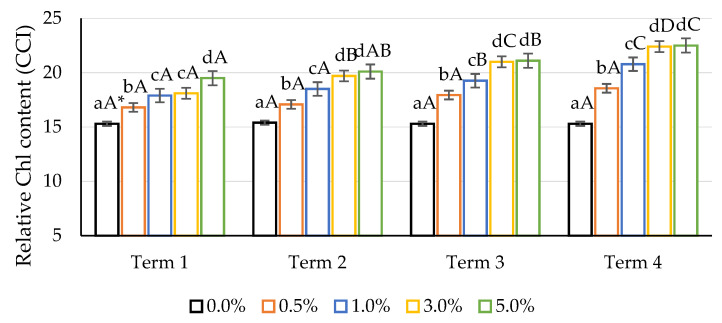
Impact of different aqueous concentrations of potassium quercetin derivative on relative Chl content (CCI) in maize leaves. * Lowercase letters indicate significant differences between the means on respective measurement times, and capital letters indicate significant differences between means on the measurement times for respective potassium quercetin derivative concentrations and exposure times (*n* = 30, *p* < 0.05).

In the conducted research, the application of an aqueous solution of quercetin derivative had a positive effect on the increase in the relative content of Chl in maize leaves (Figure 4). Regardless of the concentration applied, these values were statistically significant compared to the control. The highest concentrations—3% and 5%—had the most favourable influence on the relative content of Chl. After the use of lower concentrations, an increase in the value of the analysed parameter was also observed, but it was not as dynamic. Additionally, for the dates of measurement, an increase in quercetin concentration resulted in an increase in the relative Chl content of maize leaves. The most intense response of plants to the application of a quercetin derivative can be observed in the fourth term of measurement at the highest applied solution concentration. Statistically significant differences were noted in the case of quercetin at 1%, 3% after the second application (Terms 3 and 4); in the case of a 5% solution, the significance of the differences was already observed on the second date of measurement, which suggested that this concentration had a strong stimulating effect. The use of aqueous solutions of quercetin potassium derivative resulted in a significant increase in the content of chlorophyll, which may be due to the stimulation of chlorophyll biosynthesis and inhibition of its degradation. Moreover, the increase in chlorophyll concentration can be attributed to the more efficient removal of ROS by quercetin, and could also be due to the stabilisation of the photosynthetic reaction.

### 2.4. Chlorophyll Fluorescence

Plants growing in natural conditions are exposed to a number of unfavourable factors, generally referred to as environmental stresses, which disrupt their physiological processes and limit their growth and yield. Photosynthetic carbon assimilation is a key process in plant metabolism, and is closely related to environmental conditions. Photosynthesis consists of two main parts: photochemical processes occurring at the level of NADPH and ATP-producing thylakoid membranes, as well as the CO_2_ reduction pathway (mainly the Calvin cycle) using ATP and NADPH for CO_2_ assimilation [73]. Measurement of chlorophyll fluorescence is a non-invasive and highly sensitive method of assessing the physiological state of plants that allows for the determination of not only photosynthetic activity but also plant responses to adverse environmental conditions at an early stage, well before the appearance of visible changes [74]. This measurement helps to understand the basic mechanisms of photosynthesis and their impact on the overall assessment of the physiological state of plants. This is because photosynthesis is associated with all metabolic and physiological processes taking place in a plant cell, and any change in the environment causing changes in these processes will affect the process of photosynthesis [75,76,77,78]. A deficiency in nutrients and abiotic stresses occurring during plant vegetation directly affects the photosynthetic apparatus. Usually, a decrease in photosynthesis efficiency is the first symptom of the negative impact of stresses on a plant, which can affect the maximum PSII quantum yield and which is proportional to the F_v_/F_m_ ratio, reflecting the efficiency of light in primary photosynthetic reactions [75,79]. An imbalance in this phase leads to a loss of absorber energy and the formation of ROS in the chloroplasts. Long-term exposure of plants to stress reduces their ability to use photoenergy, thus leading to a change in photosynthesis [80], which is why it is so important to search for new, non-invasive methods for reducing abiotic stresses in field crops.

**Figure 5:**
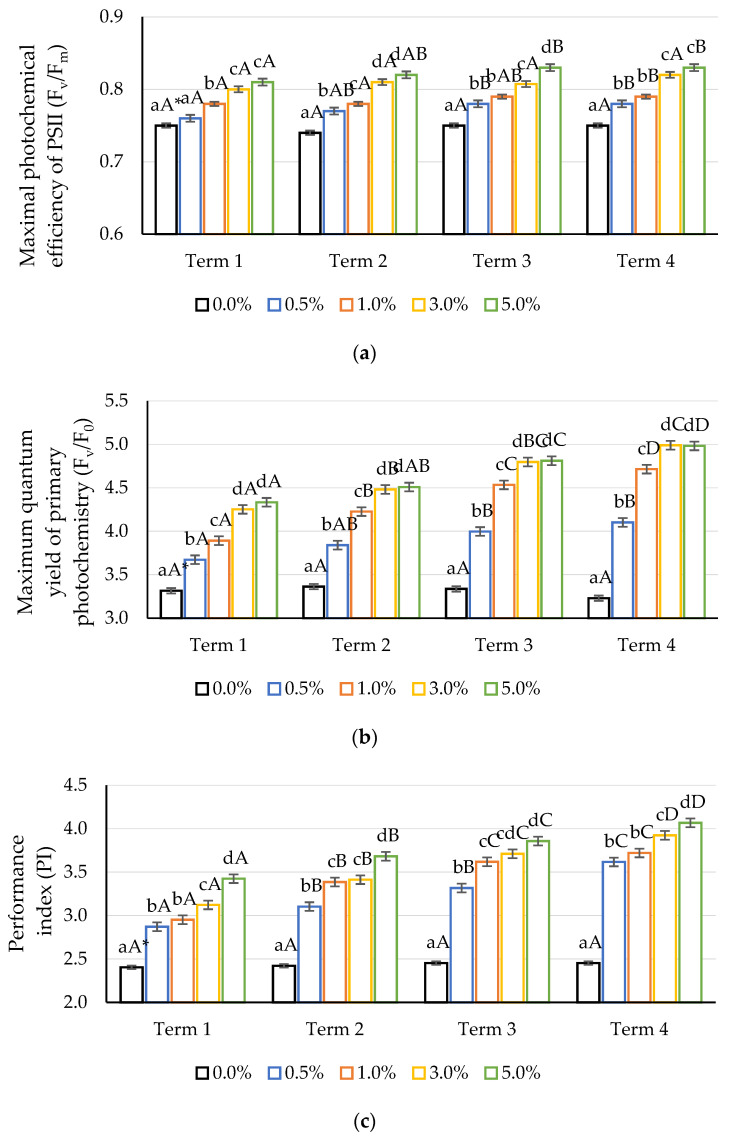
Impact of different aqueous concentrations of potassium quercetin derivative on Chl fluorescence parameters in the maize leaves: (**a**) F_v_/F_m_—maximal photochemical efficiency of PSII, (**b**) F_v_/F_0_—maximum quantum yield of primary photochemistry, (**c**) PI—performance index. * Lowercase letters indicate significant differences between the means on respective measurement times, and capital letters indicate significant differences between means on the measurement times for respective potassium quercetin derivative concentrations and exposure times (*n* = 30, *p* < 0.05).

The foliar application of an aqueous solution of a quercetin derivative stimulated the parameters of chlorophyll fluorescence in maize plants (Figure 5). The F_v_/F_m_ ratio is a measure of the light efficiency in primary photosynthesis reactions, and its value is proportional to the quantum efficiency of PSII photochemical reactions [79]. This parameter is considered a reliable measure of the photochemical activity of the photosynthetic apparatus. However, the ratio to F_v_/F_0_ is much more sensitive. It provides the same basic information but shows higher values and a higher dynamic range than F_v_/F_m_. This parameter shows a higher amplitude under stress conditions, as it immediately reflects all changes in F_v_ and/or F_0_ [81]. The maximum efficiency of the water decomposition reaction on the donor side of PSII (F_v_/F_m_) was dependent on the derivative concentration applied and the duration of the experiment. The stimulating effect was found for all analysed concentrations on each measurement date, except for the first one, in which no significant differences were noted between the plants treated with 0.5% quercetin derivative solution and the control. An intense response was observed when the 5% solution was applied on the first day after the first application (Term 1). The use of a second dose of the solution also increased the values of the discussed parameters, but they were not as dynamic.

A similar relationship was observed for the F_v_/F_m_ and the PI. The values of these parameters increased with the increase in quercetin concentration, as well as with the duration of the experiment. The F_v_/F_0_ ratio values increased significantly with increasing doses of the quercetin derivative compared to the control. As in the case of F_v_/F_m_, this parameter increased in proportion to the derivative concentration applied. The performance index describes the effective amount of energy converted by PSII. The index provides useful information on the state of the electrons by combining information on the number of active reaction centres and initial light phase reactions with data on RC electron flow [82]. In the conducted experiment, a significant difference in the PI value was observed in relation to the applied dose of the quercetin potassium derivative.

### 2.5. Total Antioxidant Capacity

The measurement of antioxidant activity (AA) can be one of the measures of stress in plants [83]. Oxidising agents trigger a cascade of biochemical reactions that allow the production of compounds that protect against their toxic effects. One such mechanism is the activation of PAL (phenylalanine ammonia lyase), which can be enhanced by the production of AA-affecting polyphenols [84]. Therefore, new natural compounds with antioxidant properties that can be used in plant production are constantly sought [85,86,87,88,89].

**Figure 6:**
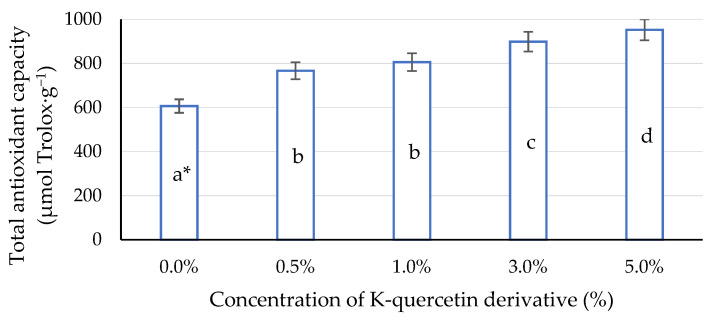
Impact of different aqueous concentrations of potassium quercetin derivative on total antioxidant capacity in maize leaves. * Lower case letters indicate significant differences between concentrations of K-quercetin derivatives (*p* < 0.05).

The total antioxidant capacity of plants increased with the increase in the quercetin concentration applied (Figure 6). Significant differences were noted between all concentrations of the quercetin derivative used in the experiment. Concentrations of 3% and 5% showed the highest total antioxidant capacity, expressed as Trolox equivalent (mg), in 100 g of maize leaves.

### 2.6. Total Phenolic Compounds

Plants have different biochemical and molecular mechanisms to combat abiotic stresses, such as antioxidant production, ion homeostasis and the accumulation of compatible solutes [90]. Several cytotoxic ROS, including superoxide (O_2_^•−^) radicals, hydrogen peroxide (H_2_O_2_), singlet oxygen (^1^O_2_) and hydroxyl (HO^•^) radicals, are regularly produced in plant cells and are detrimental to normal cell metabolism. To deal with these stresses, plants have developed a complex antioxidant defence system with enzymes such as superoxide dismutase (SOD), peroxidase (POD), catalase (CAT), polyphenol oxidase (PPO), ascorbate peroxidase (APX), guaiacol peroxidase (GPX), glutathione reductase (GR), and non-enzymatic components (ascorbate, phenolic compounds, glutathione, etc.) [91]. Depending on the tolerance and sensitivity of genotypes, differences in the expression level of antioxidant enzymes can be found [92,93]. In addition, phenolic compounds, being nucleophiles, can inhibit lipid peroxidation through their ability to remove free radicals and prevent damage caused by them [94]. This protective effect of polyphenolic antioxidants is supported by mechanistic evidence that shows that certain food-derived ingredients can prevent oxidative damage and cell apoptosis by donating electrons to unstable free radical molecules, thus neutralising their harmful effects [95,96].

**Figure 7:**
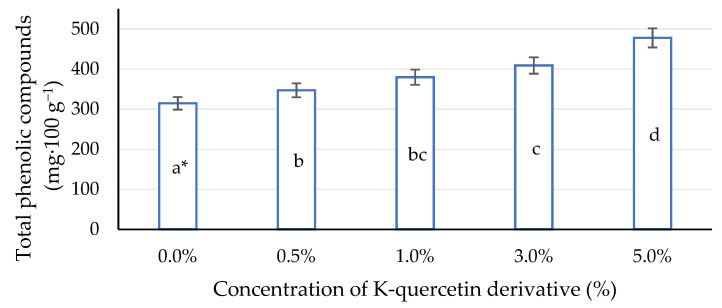
Impact of different aqueous concentrations of potassium quercetin derivative on total phenolic compounds in maize leaves. * Lower case letters indicate significant differences between concentrations of K-quercetin derivatives (*p* < 0.05).

The total content of polyphenolic compounds increased with increasing concentrations of the quercetin solution (Figure 7). The highest value of the analysed parameter was obtained for the concentration of 5%; however, significant differences were noted between all analysed concentrations of the solution and the content of polyphenolic compounds in maize leaves. Stimulating plants allows one to prevent the occurrence of harmful cellular mechanisms, hence the great interest in obtaining plant extracts with high biological activity. An important aspect is also the use of efficient and environmentally friendly extraction technologies [97,98]. However, it is also important to establish a critical value at which the effect of an action will negatively affect a given plant. In the case of quercetin, the phenomenon of the quercetin paradox is known. In living cells, this means that the antioxidant selectively targets the oxidative damage to thiol arylation. It seems that supplementation with antioxidants should consider the potential toxicity of metabolites formed during the actual antioxidant activity of free radical scavengers [32].

## 3. Materials and Methods

### 3.1. Synthesis and Physicochemical Properties of the Potassium Quercetin Derivaztive

#### 3.1.1. Determining the Optimal Stoichiometric Ratios between the Reagents

Job’s method [31] was used to determine the optimal ratios between the reagents, i.e., quercetin and potassium ions. To this end, a methanolic solution of quercetin (Sigma-Aldrich, Steinheim, Germany) at a concentration of 1 mM was added to a 1 mM KOH solution in volume ratios of 1:20–10:1, after which the absorbance was measured at the wavelength λ = 374 nm. Graphs of the relationship between the molar fraction of the reagents and the absorbance of the solutions were plotted; then, using the first derivative method, the molar fraction of the reagents, in which the absorbance value was minimal, was determined.

#### 3.1.2. Synthesis of the Potassium Quercetin Derivative

In total, 1 mmol of quercetin and 7 mmol of KOH were dissolved in 1 L of methanol. After 3 h of magnetic stirring, the solution was filtered and then concentrated and dried using a vacuum evaporator (pressure 300 mbar, temperature range 40–65 °C). The surface of the substance was ground and designated for testing.

#### 3.1.3. Analysis of Absorption Spectra in the UV-Vis Range

The quercetin derivative was reconstituted in methanol (10 µg·mL^−1^), and then the absorbance in the UV-Vis range (200–800 nm, Metash spectrophotometer) was measured with a measuring increment every 5 nm. The derivative’s absorption spectrum was compared to that of a methanolic quercetin solution.

#### 3.1.4. Analysis of Absorption Spectra in the UV-Vis Range

To 1 mL of DPPH radicals • (2,2-diphenyl-1-picrylhydrazyl, Sigma-Aldrich), at a concentration of 100 µM (in methanol), 30 µL of the quercetin derivative was added at a concentration of 10 µg·mL^−1^ (in methanol). After 30 min of incubation in the dark, the absorbance of the solutions was measured at λ = 515 nm. An aqueous solution of ABTS radicals •+ was prepared by dissolving 7 mM ABTS (2′-azobis-3-ethylbenzothiazoline-6-sulfonate, Sigma-Aldrich) in 2 mM of K_2_S_2_O_8_ solution (Chempur). After 24 h of incubation in the dark, the solution before determination was diluted to an absorbance equal to 0.7 ± 0.01, at λ = 400 nm.

To 1 mL of ABTS radicals •+ 20 µL of derivative solution was added. After 30 min of incubation in the dark, the absorbance of the solutions was measured at λ = 400 nm, against the blank. The obtained test results (DPPH •, ABTS •+) were expressed as quercetin, ascorbic acid and Trolox equivalents [99].

### 3.2. Plant Material and the Course of the Pot Experiment

The pot experiment was carried out at the University of Rzeszów (Poland). Maize seeds (*cv*. Talentro, Saatbau Linz Poland, universal use, type: F-D, FAO 260) were sown in pots (25 cm in diameter) in which 6 kg of soil with a clay sand particle size composition and a slightly acidic pH (pH: 1 M KCl 6.35; H_2_O 6.52) was placed. Experiments were carried out in four replications of 10 pots per variant (*n* = 50) in a growth chambers (Model GC-300/1000, JEIO Tech Co., Ltd., Seoul, South Korea) at a temperature of 22 ± 2 °C, a humidity of 60 ± 3% RH, a photoperiod of 16/8 h (L/D) and a maximum light intensity of about 300 μmol∙m^−2^∙s^−1^. The substrate humidity was maintained at the level of 60% of the field water capacity. The positions of the pots in the experiment were randomised weekly. Ten seeds were sown in each pot. After emergence, the plant density was set at 7 plants per pot. On the 15th and 22nd day after emergence, the maize plants were sprayed with an aqueous solution of quercetin derivative at concentrations of 0.5%, 1.0%, 3.0% and 5.0% in the amount of 50 mL per pot. Spraying was performed with a laboratory hand sprayer with a stream regulation with a dosing volume of 1.2 mL ± 0.1 during one press (outlet diameter 0.6 mm). A uniform spraying procedure was applied: the same amount of solution for each pot until the solution was completely exhausted. At the same time, deionised water was applied in the same volume in the control sample.

Measurements of the physiological processes taking place in the maize leaves (gas exchange, relative Chl content and Chl fluorescence) were performed on the first or second fully developed leaves four times: on the first and seventh days after each treatment. Then, the fresh weight of the above-ground part of the plants was harvested, and the total antioxidant capacity and total polyphenolic compounds in the maize leaves were assessed.

### 3.3. Measurement of Gas Exchange

The net photosynthetic rate (P_N_), transpiration rate (E), stomatal conductivity (g_s_) and intercellular CO_2_ concentration (C_i_) were measured on two fully developed leaves (20 measurements per concentration). The LC pro-SD photosynthesis measurement system (ADC Bioscientific Ltd., Herts, UK) was used to measure the photosynthesis of the leaves. The plant leaf photosynthesis chamber of the LCpro-SD has a flow accuracy of ±2% of its range. During the measurement, the light intensity was 300 μmol∙m^−2^∙s^−1^ and the temperature (in the measuring chamber) was 22 °C.

### 3.4. Measurement of the Relative Content of Chlorophyll

The relative chlorophyll (ChI) content was measured using a CCM-200plus handheld chlorophyll meter (Opti-Sciences, Hudson, NH, USA). Measurements were made on three fully developed leaves per pot (30 measurements per concentration).

### 3.5. Measurement of Chlorophyll Fluorescence

To measure chlorophyll fluorescence, a continuous excitation Pocket PEA fluorimeter (Pocket PEA, Hansatech Instruments, King’s Lynn, Norfolk, UK), equipped with black shading clips that were applied to the leaf lamina away from the leaf nerve, was used. The following parameters were measured: the maximum quantum yield of photosystem II (PSII) (F_v_/F_m_), the maximum quantum yield of primary photochemistry (F_v_/F_0_) and the photosynthesis yield index (PI). The fluorescence signal was collected under actinic red light, with a light source peak wavelength of 627 nm, and transmitted for 1 s at the maximum available intensity of 3500 μmol (photon) of photosynthetically active radiation (PAR) m^−2^∙s^−1^. Fluorescence measurements were performed in triplicate in each pot on the medial leaf lamina after 30 min dark adaptation (30 measurements per concentration).

### 3.6. Determination of Total Antioxidant Capacity

Total antioxidant capacity was determined using the CUPRAC method using the reduction in copper ions bound in a complex with neocuproine occurring in a neutral environment [100]. For this, 5 g of tissue was homogenised with 15 mL of 50% methanol for 30 s and then shaken for 30 min (150 rpm). The homogenate was centrifuged at 7500× *g* for 30 min, and the obtained supernatant was used for analysis. Then, 30 µL of supernatant, 50 µL of 10 mM CuCl_2_, 50 µL of 7.5 mM neocuproine, 50 µL of 1 M NH_4_Ac and 20 µL of distilled water were pipetted into the wells of the plate. The absorbance of the solutions was measured at 450 nm wavelength after 30 min of incubation in the dark. The analysis was performed three times. Total oxidising capacity was reported as the Trolox equivalent (mg) in 100 g of maize leaves.

### 3.7. Determination of the Total Content of Polyphenolic Compounds

Total polyphenolic compounds were determined using the Folin–Ciocalteu method [101]. It is based on measuring the absorbance of the complex formed in the molybdenum reduction reaction contained in the Folin–Ciocalteu reagent in an alkaline environment. Then, 30 μL of supernatant (prepared as in point 3.6), 50 μL of distilled water, 20 μL of Folin–Ciocalteu reagent and 30 μL of 20% Na_2_CO_3_ were pipetted into the wells of the plate. The absorbance of the solutions was measured at λ = 690 nm after 30 min of incubation in the dark. The results are expressed as the gallic acid equivalent (mg) contained in 100 g of maize leaves. The measurement was performed in triplicate.

### 3.8. Statistical Analysis

Statistical analysis was performed using TIBCO Statistica 13.3.0 (TIBCO Software Inc., Palo Alto, CA, USA). In order to ascertain the normality of the distribution at *p* = 0.05, the Shapiro–Wilk test was performed. The homogeneity of the variance was also checked. A repeated measures ANOVA (with time assessment as a factor) was then performed. In order to determine and verify the relationship, Tukey’s post hoc test was performed with the significance level *p* ≤ 0.05.

## 4. Conclusions

The use of an aqueous solution of a quercetin derivative improved the physiological properties and did not deteriorate the condition of maize plants, regardless of the concentration applied. Among the tested variants, compared to the control, 3% and 5% solutions had the most stimulating effect on the course of physiological processes in maize leaves. There was an increase in the values of P_N_, g_s_, CCI, F_v_/F_m_, F_v_/F_0_ and PI and a decrease in the value of C_i_. The highest total antioxidant capacity and total content of polyphenolic compounds were found for plants sprayed with a 5% solution of quercetin derivative; therefore, in this study, the optimal concentration could not be clearly selected. It is important to determine the optimal dose of a stimulant acting on a plant by taking into account the index of economic efficiency of its use. The use of a quercetin derivative at any concentration may be considered in further research for its use as a plant growth bio-stimulant. In addition, further studies are needed to determine the toxic dose (quercetin paradox) of the derivative used and to test its effectiveness on other plants, including under abiotic stresses. These results, verified in field conditions, may contribute to the development of new growth stimulants dedicated specifically to sustainable and ecological agriculture.

## Figures and Tables

**Table 1:** Antioxidant activity against ABTS and DPPH of the potassium quercetin derivative, expressed as % of the standard substance activity. * Mean ± SD.

Method	Antioxidant Activity of the Potassium Quercetin Derivative, Expressed as:		
	% of Quercetin Activity	% of Trolox Activity	% of Ascorbic acid Activity
ABTS	67.02 ± 4.21 *	104.01 ± 1.75	145.34 ± 4.56
DPPH	33.43 ± 2.26	94.12 ± 6.43	110.23 ± 4.85

## Data Availability

The data presented in this study are available on request from the corresponding author.
